# Diagnostic approach of myocarditis: strike the golden mean

**DOI:** 10.1007/s12471-013-0499-3

**Published:** 2014-01-08

**Authors:** M. R. Hazebroek, K. Everaerts, S. Heymans

**Affiliations:** 1 Department of Cardiology, Maastricht University Medical Center, Maastricht, the Netherlands; 2 Interuniversity Cardiology Institute of the Netherlands, Utrecht, the Netherlands; 3 Center for Heart Failure Research, Cardiovascular Research Institute Maastricht (CARIM), Maastricht University Medical Centre, P. Debyelaan 25, 6229 HX Maastricht, the Netherlands

**Keywords:** Myocarditis, Endomyocardial biopsy, Diagnosis

## Abstract

Myocarditis is a challenging diagnosis due to the extreme diversity of clinical manifestations. The actual incidence of myocarditis is also difficult to determine as endomyocardial biopsy (EMB), the diagnostic gold standard, is used infrequently. Nevertheless, in up to 30 % of patients with biopsy-proven myocarditis, progression to dilated cardiomyopathy (DCM) can occur and is associated with a poor prognosis. Recent position statements of the European Society of Cardiology (ESC) and the American Heart Association vary widely with regard to indications for performing an EMB in these patients. This makes decision-making, in particular for general practitioners (GPs) and regional hospitals, difficult and unclear. Therefore, we will present a short summary of the ESC Working Group on Myocardial and Pericardial Diseases statement and our suggestions for GPs and regional hospitals for the diagnostic approach in patients with suspected myocarditis.

## Introduction

Myocarditis is a challenging diagnosis due to the extreme diversity of clinical manifestations [1, 2]. The actual incidence of myocarditis is difficult to determine as endomyocardial biopsy (EMB), the diagnostic gold standard [1], is used infrequently [2]. The prognosis mainly depends on clinical presentation and EMB findings. Patients presenting with acute myocarditis and preserved left ventricular function have a good prognosis with a high rate of spontaneous improvement without sequelae [3, 4]. Interestingly, patients with fulminant viral myocarditis and haemodynamic compromise at presentation appear to have an excellent long-term prognosis and are more likely to experience complete recovery than patients with acute myocarditis [4, 5], if patients survive the acute phase supported by aggressive pharmacological and/or mechanical support. Nevertheless, in up to 30 % of patients with biopsy-proven myocarditis, progression to dilated cardiomyopathy (DCM) can occur and is associated with a poor prognosis [1, 2].

Recently, a 2013 position statement from the European Society of Cardiology (ESC) Working Group on Myocardial and Pericardial Diseases [6] recommended heart biopsy to be performed for all cases of suspected myocarditis, including most acute and chronic DCM. In contrast, the 2013 American College of **Cardiology** Foundation/American Heart Association (ACCF/AHA) [7] recommends that EMB should *not* be performed in the routine evaluation of patients with heart failure. How to find the desirable middle between these two extreme recommendations?

Here, we will present a short summary of the ESC Working Group on Myocardial and Pericardial Diseases statement and our suggestions for general practitioners and regional hospitals for the diagnostic approach in patients with suspected myocarditis.

## Definitions

In line with the statement of the ESC Working Group, we also acknowledge that there is some confusion about the terms DCM, inflammatory cardiomyopathy (CMP) and myocarditis. DCM is a clinical diagnosis based on morphological and functional characterisation of the left ventricle [1]; inflammatory CMP is both a histological and a functional diagnosis characterised by myocarditis in association with systolic and/or diastolic dysfunction [1]. Myocarditis is an inflammatory disease of the myocardium diagnosed by established histological (Dallas criteria) [8], immunological, and immunohistochemical criteria (>14 leucocytes/mm^2^ including up to 4 monocytes/mm^2^ with the presence of CD3 positive T-lymphocytes >7 cells/mm^2^) [9]. The histological diagnosis of myocarditis can be classified according to the type of inflammatory cell infiltrates: lymphocytic, eosinophilic, polymorphic, giant cell myocarditis, and cardiac sarcoidosis. The ESC task group also recommends the use of subsets of myocarditis or inflammatory cardiomyopathy:

- *Viral myocarditis*: Histological evidence for myocarditis associated with positive viral polymerase chain reaction (PCR).
- *Autoimmune myocarditis*: Histological myocarditis with negative viral PCR, with or without serum cardiac autoantibodies (AABs).
- *Viral and immune myocarditis*: Histological myocarditis with positive viral PCR and positive AABs.

## Clinical presentation

The clinical manifestations of myocarditis may range from subclinical disease to fulminant heart failure. A summary of clinical presentations of patients with biopsy-proven myocarditis is shown in Table 1.

**Table 1:** Clinical presentations of patients with biopsy-proven inflammatory heart muscle disease

1) Clinical presentations
a) Acute chest pain, percarditic, or pseudo-ischaemic
- Frequently starting with 1-4 weeks of a respiratory or gastrointestinal infection
- Frequently associated with severe and recurrent symptoms
- In the absence of angiographic evidence of CAD
b) ST/T wave changes
- ST-segment elevation or depression
- T-wave inversions
c) With or without global or regional LV and/or RV dysfunction on echocardiography or CMR
d) With or without increased TnT/TnI that may have a time course similar to acute myocardial infarction or a prolonged and sustained release over several weeks or months
2) New-onset or worsening heart failure in the absence of CAD and known causes of heart failure
a) New-onset or progressive heart failure over 2 weeks to 3 months
b) Impaired systolic LV and/or RV function, with or without an increase in wall thickness, with or without dilated LV and/or RV on echocardiography or CMR
c) Symptoms possibly started after a respiratory or gastrointestinal infection, or in the peri-partum period
d) Non-specific ECG signs, bundle branch block, AV block, and/or ventricular arrhythmias
3) Chronic heart failure in the absence of CAD and known causes of heart failure (see point 2 above)
a) Heart failure symptoms (with recurrent exacerbations) of >3 months duration
b) Fatigue, palpitation, dyspnoea, atypical chest pain, arrhythmia in an ambulant patient
c) Impaired systolic LV and/or RV function on echocardiography or CMR suggestive of DCM or non-ischaemic cardiomyopathy
d) Non-specific ECG signs, sometimes bundle branch block and/or ventricular arrhythmias and/or AV-block
4) ‘Life-threatening condition’, in the absence of CAD and known causes of heart failure comprising
a) Life-threatening arrhythmias and aborted sudden death
b) Cardiogenic shock
c) Severely impaired LV function

Caforio A et al. Current state of knowledge on aetiology, diagnosis, management, and therapy of myocarditis: A position statement of the European society of cardiology working group on myocardial and pericardial diseases. *Eur Heart J*. 2013;34:2636–2648 by permission of Oxford University Press

## Diagnosis

Several non-invasive diagnostic modalities, including cardiac magnetic resonance imaging (CMR), can be helpful in the diagnosis of myocarditis; however we emphasise that EMB remains the gold standard for the diagnosis of definite myocarditis. Nevertheless, we are aware that performing EMB is not routine practice in patients with suspected myocarditis. Therefore, we will present our suggestions regarding the first-line diagnostic steps in suspected myocarditis patients and when to refer these patients to a tertiary hospital specialised in EMB.

### First-line tests in patients with suspected myocarditis

- Electrocardiogram (ECG)
  The ECG is usually abnormal in myocarditis although ECG signs are neither specific nor sensitive [3]. Diffuse concave (rather than convex in myocardial ischaemia) ST-T segment elevations without reciprocal changes and non-specific T-wave changes are suggestive for myocarditis [10].
- Echocardiography
  Echocardiography is useful to exclude other causes of heart failure and identify ventricular thrombi; however, there are no specific echocardiographic features of myocarditis. As known, segmental or global wall motion abnormalities can mimic myocardial infarction. Patients with fulminant myocarditis tend to present with a non-dilated, thickened, and hypocontractile left ventricle, whereas patients with less acute myocarditis present with greater left ventricular dilation and normal wall thickness. Right ventricular dysfunction is uncommon but an important predictor of death or cardiac transplantation. Moreover, echocardiography is useful to monitor changes in cardiac chamber size, wall thickness, ventricular function, and pericardial effusions.
- CMR
  State-of-the-art CMR in suspected myocarditis can localise tissue injury, where different virus types seem to influence the pattern of myocardial injury [11, 12]. Moreover, CMR can quantitate tissue injury, including oedema, hyperaemia, and fibrosis, and can support the diagnosis of myocarditis (Lake Louis criteria) [11, 12]. Based on the Lake Louis criteria, when two or more of the three criteria are positive, myocardial inflammation can be predicted with a diagnostic accuracy of 78 % [11]. Timing is of crucial importance and will depend on the local availability, expertise and clinical condition of the patient. It is reasonable to perform CMR prior to EMB in clinically stable patients and it should *not* be performed in life-threatening presentations where EMB is urgently indicated [13].
- Biomarkers
  The sensitivity of cardiac biomarkers of myocardial injury varies depending on the time from symptom onset to testing and the cut-off values used [6].
  Erythrocyte sedimentation rate and C-reactive protein levels are often elevated in myocarditis, but they do *not* confirm the diagnosis. Cardiac troponins are more sensitive than creatine kinase levels, however are also non-specific and when normal do *not* exclude myocarditis. The same is true for cardiac hormones such as brain natriuretic peptides, circulating cytokines, and markers related to extracellular matrix degradation. Furthermore, viral serology is of limited utility in the diagnosis of viral myocarditis due to a high prevalence of circulatory IgG antibodies to cardiotropic viruses in the general population without viral heart disease. Serum cardiac AABs to various cardiac and muscle-specific autoantigens can be of diagnostic and prognostic value, however are not commercially available and/or validated yet.

## New proposed criteria for clinically suspected myocarditis

Myocarditis should be suspected in the presence of:

- One or more of the clinical presentations in Table 1, with or without ancillary features
*And*
- One or more of the diagnostic criteria from different categories (I to IV) in Table 2
*Or*
- When the patient is asymptomatic, 2 or more diagnostic criteria from different categories (I to IV)

**Table 2:** Diagnostic criteria for clinically suspected myocarditis

Clinical presentations
- Acute chest pain, percarditic, or pseudo-ischaemic
- New onset (days up to 3 months) or worsening of: dyspnoea at rest or exercise, and/or fatigue, with or without left and/or right heart failure signs
- Subacute/chronic (>3 months) or worsening of: dyspnoea at rest or exercise, and/or fatigue, with or without left and/or right heart failure signs
- Palpitations, and/or unexplained arrhythmia symptoms and/or syncope, and/or aborted sudden cardiac death
- Unexplained cardiogenic shock
Diagnostic criteria
1. ECG/Holter/stress test features
Newly abnormal 12 lead ECG and/or Holter and/or stress testing, any of the following: I to III degree atrioventricular block, or bundle branch block, ST/T wave change (ST elevation or non ST elevation, T wave inversion), sinus arrest, ventricular tachycardia or fibrillation and asystole, atrial fibrillation, reduced R wave height, intraventricular conduction delay (widened QRS complex), abnormal Q waves, low voltage, frequent premature beats, supraventricular tachycardia
2. Myocardiocytolysis markers
Elevated TnT/TnI
3. Functional and structural abnormalities on cardiac imaging (echo/angio/CMR)
New, otherwise unexplained LV and/or RV structure and function abnormality (including incidental finding in apparently asymptomatic subjects): regional wall motion or global systolic or diastolic function abnormality, with or without ventricular dilatation, with or without increased wall thickness, with or without pericardial effusion, with or without endocavitary thrombi
4. Tissue characterisation by CMR
Oedema and/or LGE of classical myocarditic pattern^9^

Caforio A et al. Current state of knowledge on aetiology, diagnosis, management, and therapy of myocarditis: A position statement of the European society of cardiology working group on myocardial and pericardial diseases. *Eur Heart J*. 2013;34:2636–2648 by permission of Oxford University Press

Our suggestions include that all patients with clinically suspected myocarditis should be admitted and considered for selective coronary angiography, in line with the recommendations from the ESC Working Group. We differ from the perspective of both the ESC and ACCF/AHA regarding the indication for EMB in patients with suspected myocarditis. We recommend the golden mean between both statements and use a more practical approach, particularly for patients presenting to GPs and/or regional hospitals that often have no access to state-of-the-art CMR or cannot safely perform EMB. Therefore, referral for EMB in acute suspected myocarditis patients (point 1, 2 & 4 in Table 1) is recommended in the case of:

- A life-threatening arrhythmia
- LV dysfunction that does not improve 4–5 days after onset of symptoms
- LV dysfunction that progressively deteriorates within 4–5 days after onset of symptoms
- Recurrent myocarditis

## Clinical management and outcome

Treatment strategies consist of both conventional therapy and in case of performing an EMB aetiology-based treatment. Conventional treatment consists of ACE inhibitors, angiotensin II receptor blockers, aldosterone antagonists and beta-blockers in case of heart failure, according to the ESC guidelines for heart failure. In line with these recommendations, both ACE inhibitors and beta-blockers were independently associated with LV function recovery in biopsy-proven acute myocarditis patients with LVEF <50 % [4]. Although there is a lack of large multicentre trials investigating aetiology-based treatment, several strategies can be considered based on expert consensus. These include immunomodulatory therapy (antiviral therapy, high-dose intravenous immunoglobulins, immunoadsorption) or immunosuppressive therapy (in virus-negative and AAB-positive myocarditis). Serial EMBs are recommended to evaluate the effect of these aetiology-based treatment strategies and should be modified accordingly. Notably, all recurrences of suspected myocarditis should be treated as the initial episode, wherein EMB is highly recommended to investigate a possible cause.

In general, approximately 50 % of patients presenting with acute myocarditis will spontaneously improve without sequelae within 2–4 weeks; however about 25 % will develop persistent cardiac dysfunction and up to 25–30 % may deteriorate and either die or progress to end-stage DCM with the need for heart transplantation (HTx) [1–4]. Concordantly, in a recent large study of biopsy-proven acute myocarditis patients, the mortality rate after a mean of 12 years of follow-up was 30 % and 10 % underwent HTx [4]. Of note, events still occurred after 8 years of follow-up, particularly in those patients presenting with rhythm disturbances [4]. Finally, EMB evidence of giant cell myocarditis has the highest 1-year mortality and HTx rate of ~70 %, with recurrences in up to 25 % in the allograft heart [14]. Therefore, unless otherwise indicated, long-term non-invasive cardiological follow-up is recommended in all patients who have experienced myocarditis [6].

## Conclusion

The diagnosis of myocarditis remains challenging and the exact approach varies, as reflected by the different recommendations of both the ESC and ACCF/AHA. Particularly in the setting of GPs or regional hospitals, timing of referral of a suspected myocarditis patient to a tertiary hospital remains unclear. Therefore, we have suggested a more practical approach for this matter in order to standardise referral of suspected myocarditis patients and improve patient care. Nevertheless, multicentre randomised trials are needed to provide further insight in aetiology-based treatments which may improve outcome in these predominantly young patients.
